# Correlation between body composition and biomechanical measurements of performance for mixed martial arts athletes – a pilot study

**DOI:** 10.1186/1550-2783-11-S1-P28

**Published:** 2014-12-01

**Authors:** Lacy M Puttuck, Michael S Palmieri

**Affiliations:** 1 The Institute of Sport Science & Athletic Conditioning, Las Vegas, Nevada, USA

## Background

Mixed Martial Arts is a weight-category dependent sport, and its athletes often attempt to maintain a body weight that is relatively close to their fighting class. However, they frequently experience moderate to high levels of training intensity, long term lack of recovery, and a habitual state of nutritional deficit as part of their training, fight prep, and competition. As a result, their body fat percentages remain relatively low (6-13%); however, they must also be able to retain a relatively high level of certain biomechanical capacities in order to maintain their competitiveness in the sport. If they are not capable of accomplishing both variables successfully, a marked decrease in their performance is likely.

## Methods

This pilot study examined how two biomechanical measurements of performance (Force Production and Anaerobic Tolerance) correlated to Body Composition in experienced male MMA athletes that volunteered to participate in this study. The athletes’ training regimens consisted on averaged of two hours per day, five days per week of moderate to high intensity exercise, and included the following sport-specific related skills: Muai Thai; Brazilian Ju Jitsu; MMA sparring; strength and conditioning training. Nutrition protocol included the following general parameters: 50-60% of calories from CHO; 0.75-1 gram protein per lb of body weight; 20-25% of calories from lipid; adequate hydration of at least 1 gallon of water per day. Tests conducted consisted of a deadlift until failure, a 300 yard shuttle run, and Caliper/circumference measurements. A force plate was employed during the deadlift to measure Force Production; the shuttle run was timed to account for Anaerobic Tolerance; and the measurements determined Body Composition using the Jackson / Pollock seven site method. Consent to publish the results was obtained from all participants.

## Results

A correlation analysis, assessing the relationship between two Biomechanical Measures and Body Fat Percentage, determined a positive correlation (R2 = 0.65) between Force Production and Body Fat Percentage. In addition, an even stronger positive correlation (R2 = 0.77) existed between Anaerobic Tolerance and Body Fat percentage.

## Conclusion

This pilot study found that certain biomechanical measures for performance, in particular, Force Production and Anaerobic Tolerance, are consistently correlated with a body fat between 6 and 13 percent for experienced, male MMA athletes. As these variables are closely related to the performance of these athletes, the results of this pilot study demonstrate the need for a study that investigates how these biomechanical measures, and others, may be improved upon, while at the same time, maintaining a body fat percentage advantageous to the athletes.

